# Closely related octopus species show different spatial genetic structures in response to the Antarctic seascape

**DOI:** 10.1002/ece3.3327

**Published:** 2017-09-05

**Authors:** Jan M. Strugnell, A. Louise Allcock, Phillip C. Watts

**Affiliations:** ^1^ Centre for Sustainable Tropical Fisheries and Aquaculture Marine Biology and Aquaculture James Cook University Townsville Qld Australia; ^2^ Department of Ecology, Environment and Evolution School of Life Sciences La Trobe University Melbourne Vic. Australia; ^3^ Ryan Institute and School of Natural Sciences National University of Ireland Galway Galway Ireland; ^4^ Department of Ecology and Genetics University of Oulu Oulu Finland

**Keywords:** Antarctica, octopus, microsatelliteisolation by depth, Southern Ocean

## Abstract

Determining whether comparable processes drive genetic divergence among marine species is relevant to molecular ecologists and managers alike. Sympatric species with similar life histories might be expected to show comparable patterns of genetic differentiation and a consistent influence of environmental factors in shaping divergence. We used microsatellite loci to quantify genetic differentiation across the Scotia Arc in three species of closely related benthic octopods, *Pareledone turqueti*,* P. charcoti,* and *Adelieledone polymorpha*. The relative importance of environmental factors (latitude, longitude, depth, and temperature) in shaping genetic structure was investigated when significant spatial genetic structure was uncovered. Isolated populations of *P. turqueti* and *A. polymorpha* at these species’ range margins were genetically different to samples close to mainland Antarctica; however, these species showed different genetic structures at a regional scale. Samples of *P. turqueti* from the Antarctic Peninsula, Elephant Island, and Signy Island were genetically different, and this divergence was associated primarily with sample collection depth. By contrast, weak or nonsignificant spatial genetic structure was evident across the Antarctic Peninsula, Elephant Island, and Signy Island region for *A. polymorpha*, and slight associations between population divergence and temperature or depth (and/or longitude) were detected. *Pareledone charcoti* has a limited geographic range, but exhibited no genetic differentiation between samples from a small region of the Scotia Arc (Elephant Island and the Antarctic Peninsula). Thus, closely related species with similar life history strategies can display contrasting patterns of genetic differentiation depending on spatial scale; moreover, depth may drive genetic divergence in Southern Ocean benthos.

## 1 INTRODUCTION

The marine environment presents various physical features that can affect population connectivity, such as deep water (Baums, Boulay, Plato, & Hellberg, 2012; Knutsen, Jorde, Bergstad, & Skogen, 2012), currents, fronts, and gyres (Galarza et al., 2009; Galindo, Olson, & Palumbi, 2006; Young et al., 2015). In addition, dispersal may be constrained by intrinsic factors; for example, physiological constraints will dictate the ability to traverse gradients in temperature or salinity (Johannesson & André, 2006; Sotka, Wares, Barth, Grosberg, & Palumbi, 2004), while the distribution of suitable habitat or prey preferences may limit dispersal in some taxa (Cowan & Sponaugle, 2009; Rocha, Bass, Robertson, & Bowen, 2002). Nonetheless, barriers to dispersal in marine environments typically are less obvious than those in terrestrial and freshwater environments where the effect of many landscape features that potentially affect dispersal can be relatively well studied (Micheletti & Storfer, 2015; Van Strien, Keller, & Holderegger, 2012), often in multiple species (e.g., Hayes & Sewlal, 2004; Von Oheimb et al., 2013).

Using dispersal models is an apparently convenient method to identify potential barriers in the marine environment. Indeed, several studies on marine species have found reasonable congruence to the level of connectivity among populations inferred by oceanographic modeling and analysis of population genetic data (Galindo et al., 2006; Young et al., 2015). However, creating realistic oceanographic models to simulate dispersal for many marine species remains challenging for several reasons, particularly the comparatively limited biological and physical data that are available for most marine environments. Moreover, available data are likely confounded by seasonal and interannual oceanographic variation and inadequate knowledge about target species’ life histories (e.g., data on timing and duration of spawning, fecundity, and mortality rates). In remote areas, such as the Southern Ocean, where obtaining relevant seascape and biological data is impractical, an alternative strategy to identify the presence of major dispersal barriers is to quantify spatial genetic structure in multiple species simultaneously.

Identifying the pathways and barriers to dispersal and then determining which features affect several species and which features are species‐specific are central in understanding the processes that shape the rate of adaptation and speciation. For example, species‐specific barriers would be suggestive of intrinsic responses to the landscape, while concordant breaks in spatial genetic structure across multiple taxa point toward a general lack of genetic exchange. Identifying these specific‐ versus general‐dispersal boundaries can be used to inform spatial resource management (e.g., design of marine protected areas) (Cowan & Sponaugle, 2009).

High latitude marine areas, and notably the Southern Ocean, are particularly poorly studied in terms of the potential dispersal routes and barriers to benthic marine species. Most studies in the Southern Ocean have examined spatial genetic structure at a broad scale, with an emphasis on the degree to which the fast flowing Antarctic Circumpolar Current (ACC) limits dispersal between populations from South America and populations situated on the Antarctic continent/sub‐Antarctic (e.g., Hunter & Halanych, 2008; Thornhill, Mahon, Norenburg, & Halanych, 2008; Wilson, Schrödl, & Halanych, 2009). A lack of understanding about spatial genetic structure at finer scales limits the extent to which we could, for example, designate effective protected areas or model a potential change in distributions in response to climate change.

The Scotia Arc region of the Southern Ocean represents an ideal area to investigate patterns of genetic structure across multiple species. Spatial genetic structure in the island arc region is expected to be complex: This region is subject to strong currents (due to its location within the course of the ACC), it is subject to a range of temperatures with different regions being influenced by the Pacific, Atlantic, and Southern Oceans, the ocean depths are variable (in excess of 4 km in places), and the Arc comprises several island groups of both continental and volcanic origin. In addition, the Scotia Sea has experienced rapid increases in ocean temperatures recently (Meredith & King, 2005; Whitehouse et al., 2008) making it an important location for investigating the impact of climate change. Oceanographic modeling indicates a predominantly unidirectional flow of water from the Antarctic Peninsula in a northeast direction toward Elephant Island, the South Orkney Islands and then toward the islands of South Georgia and Shag Rocks (Young et al., 2015); however, high resolution seascape data are not readily available for a formal landscape study of this area.

Studies on several marine invertebrates across the Scotia Arc region indicate that deepwater channels and the major currents between South Georgia and other sub‐Antarctic islands and the Antarctic continent drive substantial population genetic differences between these locations (González‐Wevar, Saucède, Morley, Chown, & Poulin, 2013; Hoffman, Peck, Linse, & Clarke, 2011; Hunter & Halanych, 2008; Krabbe, Leese, Mayer, Tollrian, & Held, 2010; Strugnell, Watts, Smith, & Allcock, 2012; Wilson, Hunter, Lockhart, & Halanych, 2007). Also, populations of direct developing (*Margarella antarctica*) gastropods around Signy Island are genetically different to those off the Antarctic Peninsula, as are populations of broadcast spawning (*Nacella concinna*) gastropods, with deepwater channels between these areas proposed to limit adult and larval movement, respectively (Hoffman, Clarke, Clarke, Fretwell, & Peck, 2011; Hoffman, Clarke, Linse, & Peck, 2011; Hoffman, Peck, et al., 2011). In addition, passive dispersal during the early life history stages can shape population genetic structure in Antarctic fishes. Model projections predicted that stronger genetic differentiation would occur in *Champsocephalus gunnari* (with a three‐month larval phase) than in *Notothenia rossii* (whose larval phase is estimated to be more than six months); this prediction was confirmed using microsatellite analyses (Young et al., 2015). Nonetheless, no study has yet quantified the congruence of fine scale barriers to dispersal across closely related marine species that inhabit the Scotia Arc region.

The Southern Ocean benthic octopods represent an ideal lineage to investigate whether congruent barriers to dispersal exist because they are benthic taxa that possess large eggs, which likely hatch as benthic young. It is well accepted that octopuses with eggs above a certain size possess crawl away young (see Boletzky, 1974). Given limited dispersal by adults and larvae, significant spatial genetic structure in response to the varied landscape of the Scotia Arc is expected. Three closely related octopus species can be caught in large numbers across the Scotia Arc region. *Pareledone turqueti* (Joubin, 1905) is distributed across the Scotia Arc including the waters surrounding South Georgia and Shag Rocks. This species is circumpolar in its distribution and inhabits waters <1,116 m in depth. *Adelieledone polymorpha* (Robson, 1932) is distributed across the Scotia Arc but is absent from Shag Rocks. It occurs in waters <1,510 m in depth and also has a circumpolar distribution. *Pareledone charcoti* (Joubin, 1905) has a more restricted distribution than the other two species and is found off the northern Antarctic Peninsula and the South Shetland Islands (Allcock, 2005) in waters <286 m in depth; this species apparently has a restricted range as it is unknown from the waters to the east of Elephant Island including South Georgia, Shag Rocks, and the South Orkney Islands.

The aim of this study was to identify whether different octopus species, with putative similar life histories, share comparable levels of genetic diversity and spatial genetic structure across the Scotia Arc.

## 2 MATERIALS AND METHODS

### 2.1 Specimen collection

**Figure 1:**
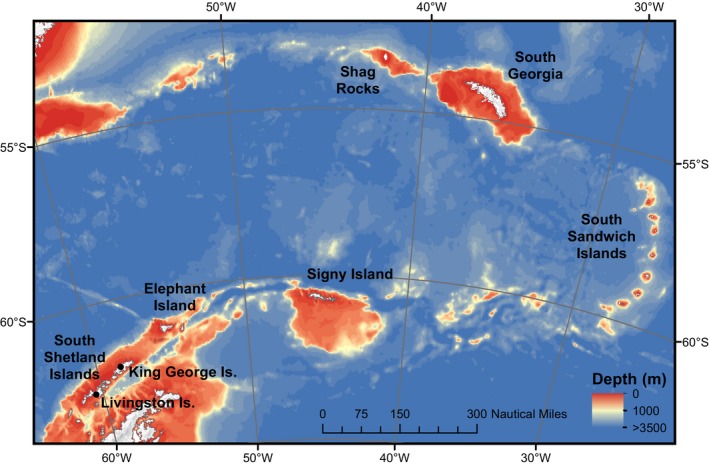
Map of the Scotia Arc indicating the sample sites for *Adelieledone polymorpha*,* Pareledone charcoti,* and *Pareledone turqueti* and the main geographic regions mentioned in the text

Samples were collected from the Scotia Arc using benthic trawls (Agassiz and otter trawls) between 62 and 666 m depth between 1987 and 2009. *Adelieledone polymorpha* was collected from the South Shetland Islands (*n *= 68), Elephant Island (*n *= 108), Signy Island (*n *= 2), and South Georgia (*n *= 111) (Figure 1). *Pareledone charcoti* was collected from the South Shetland Islands (*n *= 11) and Elephant Island (*n *= 350) (Figure 1). *Pareledone turqueti* was collected from the South Shetland Islands (King George Island [*n *= 11], Livingston Island [*n *= 35]), Elephant Island (*n *= 93), Signy Island (*n *= 9), South Georgia (*n *= 182), Shag Rocks (*n *= 125), and the South Sandwich Islands (*n *= 1) (Figure 1, Table 1). The mean depth from which *P. charcoti* was collected at Elephant Island (111 m) was shallower than that for *A. polymorpha* (287 m) and *P. turqueti* (227 m) (Table 1). Similarly, *P. charcoti* was collected from shallower mean depths at the Peninsula (117 m) than *A. polymorpha* (294 m) while *P. turqueti* was collected in greater numbers in deeper waters (544 m) at this location. At South Georgia, *A. polymorpha* (192 m) and *P. turqueti* (194 m) were collected from comparable depths. Latitude, longitude, and depth were recorded for each sampling event. Temperature was recorded from most (78%) sampling locations via CTDs (Conductivity, Temperature, and Depth) that were deployed just prior to each trawling event. Tissue samples were stored in 70–95% ethanol at −20°C, prior to preserving whole animals in formalin for taxonomic identification (by ALA).

**Table 1:** Summary of depth data (in metres) for *Adelieledone polymorpha, Pareledone charcoti,* and *Pareledone turqueti*

	*P. charcoti*		*A. polymorpha*			*P. turqueti*				
	Elephant Island	Peninsula	Elephant Island	Peninsula	South Georgia	Elephant Island	Peninsula	Signy Island	South Georgia	Shag Rocks
*N*	350	11	108	68	111	93	46	9	182	125
Min.	62	94.9	89.2	94	102	95.28	94.9	218.64	100	112
Max.	286.1	175.6	486.6	666	304	486.6	804	504.74	420	533
Mean	110.9	116.91	286.8	293.57	191.79	227.31	544.41	296.01	193.77	182.55
SD	30.54	37.69	88.41	96.93	53.86	102.18	259.64	118.54	73.28	86.19

Min., minimum depth; Max., maximum depth; Mean, mean depth; SD, standard deviation. All values are in metres. See Figure 1 for sample locations.

### 2.2 Microsatellite genotyping

Samples were genotyped at ten of the microsatellite loci described for *P. turqueti* (Strugnell, Allcock, & Watts, 2009a; Strugnell et al., 2012), ten of the loci described for *P. charcoti* (Strugnell et al., 2009a), and nine of the loci described for *A. polymorpha* (Strugnell, Allcock, & Watts, 2009b) (Appendix S1, Supporting information) using published PCR conditions (Strugnell et al., 2009a,b). PCR products were pooled into genotyping panels along with GENESCAN‐500 size standard (Applied Biosystems) and separated by capillary electrophoresis on an ABI3130xl (Applied Biosystems). Allele sizes were determined using GENEMAPPER v.3.0 (Applied Biosystems).

### 2.3 Data analyses

MICROCHECKER v.2.2.3 (Van Oosterhout, Hutchinson, Wills, & Shipley, 2003) was used to check data for the presence of null alleles, the frequencies of which were estimated using FREENA (Chapuis & Estoup, 2007). Where significant frequencies of null alleles were detected, false homozygote frequencies were used to adjust the number of null alleles per sample (Sun, Lian, Navajas, & Hong, 2012). GENEPOP v.4.1.3 (Raymond & Rousset, 1995; Rousset, 2008) and FSTAT v.2.9.3 (Goudet, 2001) were used to provide descriptive statistics: departures from expected Hardy–Weinberg Equilibrium (HWE) conditions, extent of linkage disequilibrium (LD) between loci, numbers of private alleles, allelic richness (*A*_R_), observed (*H*_o_) and expected heterozygosity (*H*_e_) and the amount of genetic differentiation (Wright's [1951] *F*_ST_) among pairs of large (n >* *10) samples. The significance of estimates of pairwise *F*_ST_ from zero was assessed through 2,000 permutations of genotypes between populations. Significance of multiple tests was adjusted (α=0.05) using a sequential Bonferroni correction (Rice, 1989). Given differing sample sizes and numbers of loci between each species, the statistical power of each microsatellites panels to detect true levels of population differentiation (*F*_ST_) was evaluated using the POWSIM software (Ryman & Palm, 2006). We computed the locations of five barriers (areas that represented the strongest genetic differences) among samples of *P. turqueti* using BARRIER v. 2.2 (Manni, Guérard, & Heyer, 2004). Barriers were calculated using matrices of pairwise *F*_ST_ (adjusted for null alleles as described above), with the robustness of the barriers estimated by resampling 100 bootstrapped matrices. Bootstrapped matrices of *F*_ST_ were calculated using the writeBoot function in the R‐package DiveRsity (Keenan, McGinnity, Cross, Crozier, & Prodöhl, 2013).

Spatial genetic structure was quantified using the Bayesian model‐based clustering approach implemented in STRUCTURE v.2.3.4 (Pritchard, Stephens, & Donnelly, 2000) that simultaneously identifies populations (clusters) and assigns individual genotypes to these model populations. Five independent runs of STRUCTURE were completed for each species, with a burn‐in of 20,000 that was followed by 200,000 Monte Carlo Markov chain replicates and a search for the number of clusters (*K*) between 1 and 15; the admixture model and correlated allele frequencies were used. STRUCTURE HARVESTER v.0.6.92 (Earl & von Holdt, 2012) was used to determine the most pronounced level of population subdivision using the method of Evanno, Regnaut, and Goudet (2005). CLUMPP v.1.1.2 (Jakobsson & Rosenberg, 2007) was used to summarize data from replicate STRUCTURE runs, and DISTRUCT v.1.1 (Rosenberg, 2004) was used to display results.

To assess possible source‐sink population structure, we estimated the directional relative migration between sample locations (Sundqvist, Zackrisson, & Kleinhans, 2013). Briefly, the genetic composition of a potential pool of migrants was calculated as the geometric means of the allele frequencies of a pair of samples; next, the level of genetic differentiation (e.g., D, Jost, 2008) between each population and the migrant pool was used to provide two directional estimates of genetic differentiation. This procedure is repeated for all pairs of samples, and the concomitant directional measures of genetic differentiation are used to calculate the directional relative migration among all sample pairs (Sundqvist et al., 2013). Directional relative migration was calculated using the function divMigrate within the R‐package diveRsity (Keenan et al., 2013). Bootstrapping was used (1,000 iterations) to generate 95% confidence intervals and to determine whether migration is significantly higher in one direction than the other.

We used GESTE v.2.0 (Foll & Gaggiotti, 2006) to determine whether environment variation explains spatial genetic structure in *P. turqueti* (there was insufficient genetic structure for analysis of *P. charcoti* and *A. polymorpha*). GESTE uses a generalized linear model to relate environmental factors to a population‐specific value of genetic differentiation (*F*_ST_). Environmental factors included in the analysis were as follows: latitude, longitude, depth, and temperature (from CTD data). Ten pilot runs were used (length = 5,000) to determine acceptance rates for parameters of the Monte Carlo Markov Chain. Subsequently, the analysis was run with a burn‐in of 50,000, a sample size of 10,000 and a thinning interval of 20.

## 3 RESULTS

### 3.1 Genetic diversity

We genotyped 289 samples of *Adelieledone polymorpha*, 361 samples of *P. charcoti,* and 456 samples of *Pareledone turqueti* from locations across the Scotia Arc (Figure 1).

Just six (of 54 tests over all samples) pairs of loci for *P. charcoti*, eight (of 108 tests) pairs of loci for *A. polymorpha,* and nine (of 254 tests) pairs of loci for *P. turqueti* had significant LD after correction for multiple testing within each of the large sample regions (Tables S4–S6, Strugnell et al., 2012). As no locus pair was out of LD across several samples, all loci were retained for analyses. Most sample‐locus comparisons met expected HWE conditions (37 of 52 tests) (Supporting Information), with the majority of those tests that did not meet HWE conditions from Elephant Island (11 of 15 tests).

Genetic diversity differed among species and among locations. Thus, expected heterozygosities (*H*_e_) were lowest for *P. charcoti* (*H*_e_ < 0.40 at both locations), intermediate for *A. polymorpha* (*H*_e_ = 0.54–0.58 and highest for *P. turqueti* (*H*_e_ > 0.69 at all locations) (Table 2). Expected heterozygosities were lower in the Islands of South Georgia than other locations for *Adelieledone polymorpha* and *P. turqueti*. The Antarctic Peninsula region and Elephant Island had the highest *H*_e_ for *P. charcoti* and *P. turqueti,* respectively, and both of these locations had high expected heterozygosities for *A. polymorpha* (Table 2). Elephant island samples had a high proportion of private alleles for all three species (*P. charcoti* 80%, *A. polymorpha* 33%, *P. turqueti* 19%), although South Georgia samples had the highest proportion of private alleles for *A. polymorpha* (40%). Shag Rocks had the highest proportion of private alleles for *P. turqueti*, although this was only slightly higher than for South Georgia and Elephant Island (21%, 20%, and 19%, respectively) (Table 2).

**Table 2:** Sample sizes and summary genetic diversity statistics for samples of *Pareledone charcoti*,* Adelieledone polymorpha,* and *Pareledone turqueti* genotyped at 9, 10, and 10 loci, respectively (nulls corrected)

	*P. charcoti*		*A. polymorpha*			*P. turqueti*				
	Elephant Island	Peninsula	Elephant Island	Peninsula	South Georgia	Elephant Island	Peninsula	Signy Island	South Georgia	Shag Rocks
*N*	350	11	108	68	111	93	46	9	182	125
*N*_A_	36.5	7.5	18.78	14.56	18.44	34.1	25	8.1	38	30.2
*N*_PA_	29.3	0.4	6.11	3.44	7.44	6.6	3.9	0.6	7.7	6.3
*A*_R_	5.56	5.95	1.64	1.62	1.67	10	9.7	‐	9.5	8.4
*H*_O_	0.348	0.382	0.534	0.561	0.536	0.760	0.733	0.744	0.684	0.744
*H*_E_	0.382	0.393	0.573	0.578	0.535	0.743	0.733	0.738	0.694	0.730

*N*, number of individuals per population; *N*_A_, average number of alleles across all loci per population; *N*_PA_, average number of private alleles across all loci per population; *A*_R_, average allelic richness across all loci per population (standardized to five individuals for *P. charcoti*, one individual for *A. polymorpha*, nine for *P*. *turqueti*); *H*_O_, observed level of heterozygosity; *H*_E_, expected level of heterozygosity.

See Figure 1 for sample locations. Small samples from Signy Island for *A. polymorpha* (*n *= 2) and South Sandwich Islands for *P. turqueti* (*n *= 1) are not included.

### 3.2 Spatial structure

**Figure 2:**
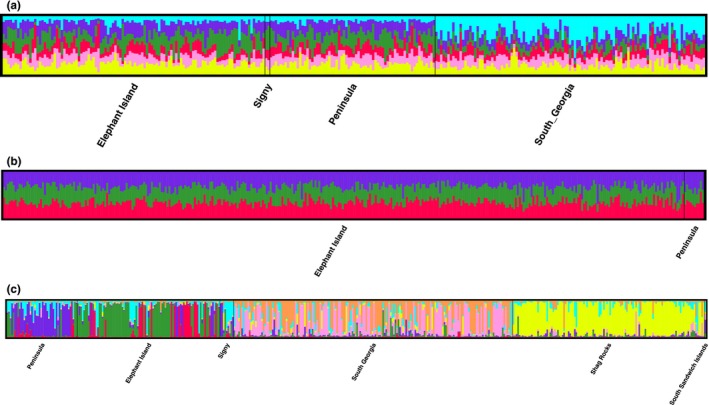
STRUCTURE assignment of individuals across all populations into clusters of best fit at (a) K = 6 (*Adelieledone polymorpha*), (b) K = 3 (*Pareledone charcoti*), (c) K = 7 (*Pareledone turqueti*). Colors indicate percentage contribution of individuals to assigned clusters (y axis), individuals represented by each line (x axis). Black lines separate populations from which individuals belong

STRUCTURE analyses identified *K *=* *6 for *A. polymorpha*,* K *=* *3 for *P. charcoti* and *K *=* *7 for *P. turqueti* (Figure 2), with the level of association between these clusters and the geographic locations of the samples depending upon the species and the spatial scale.

Both *A. polymorpha* and *P. turqueti* showed a similar pattern of spatial genetic structure at a large geographic scale, with apparent isolation of populations around South Georgia and all other localities (Figure 2, Table 3)*. Pareledone turqueti* also exhibited substantial spatial structure between Shag Rocks and all other localities (Figure 2, Table 3) but neither *A. polymorpha* and *P. charcoti* inhabit the waters surrounding Shag Rocks. Greatest genetic differentiation (*F*_ST_ **=* *0.052) for *A. polymorpha* occurred between South Georgia and the Peninsula, whilst comparable values of *F*_ST_ occurred between South Georgia and Signy Island (*F*_ST_ **=* *0.065) and South Georgia and Shag Rocks for *P*. *turqueti* (*F*_ST_ **=* *0.060). Accordingly STRUCTURE analysis identified a distinct cluster corresponding to the location of South Georgia for both of these species (Figure 2). For example, in *A. polymorpha* genetic differences, as estimated by the average proportions of membership to model clusters (*Q*), were evident between South Georgia (Q_2_ **=* *0.11, Q_3_ **=* *0.14, Q_6_ **=* *0.31) and all other locations (Elephant Island Q_2_ **=* *0.23, Q_3_ **=* *0.20, Q_6_ **=* *0.10; Peninsula Q_2_ **=* *0.22, Q_3_ **=* *0.20, Q_6_ **=* *0.09) for three of the clusters (Table S13). Likewise, STRUCTURE estimated samples of *P. turqueti* from both (1) Shag Rocks (Q_5_ **=* *0.76) and (2) South Georgia (Q_4_ **=* *0.37, Q_7_ **=* *0.38) to be genetically different to samples from other locations (Table S15). Consistent with these results, the principal barrier for *P. turqueti* separated samples from (1) South Georgia and Shag Rocks and (2) other locations (Supporting Information).

**Table 3:** Variation in genetic differentiation (*F*_ST_) among sample locations in the Southern Ocean for three species of octopus, as measured using microsatellite loci

	Peninsula (East)	Peninsula (West)	Elephant Island	Signy Island	South Georgia	Shag Rocks
*Pareledone charcoti*						
Antarctic Peninsula		–	NS			
Elephant Island		0.0089	–			
*Adelieledone polymorpha*						
Antarctic Peninsula		–	0.0083		0.0083	
Elephant Island		0.0055	–		0.0033	
South Georgia		0.0523	0.0462		–	
*Pareledone turqueti*						
WAP	–	NS	0.0033	0.0033	0.0033	0.0033
EAP	0.0097	–	NS	NS	0.0033	0.0033
Elephant Island	0.0307	0.0101	–	0.0033	0.0033	0.0033
Signy Island	0.0514	0.0484	0.0552	–	0.0033	0.0033
South Georgia	0.0517	0.0448	0.0346	0.0650	–	0.0033
Shag Rocks	0.0748	0.0535	0.0579	0.0902	0.0597	–

Significance after Bonferroni correction above diagonal. *F*_ST_ below the diagonal.

*p* values obtained after: 20, 120, 300 permutations (*P. charcoti*,* A. polymorpha*, and *P. turqueti,* respectively). Indicative adjusted nominal level (5%) for multiple comparisons is 0.05, 0.008333.

At a regional scale, there were notable differences in the pattern of genetic differentiation among the three octopus species (Figure 2, Table 3). Thus, genetic differentiation between Elephant Island and the Peninsula was low (*F*_ST_ **=* *0.0089) and not significantly different from 0 for *P. charcoti* (Figure 2, Table 3), consistent with the STRUCTURE output that revealed no distinct clusters of *P. charcoti* individuals associated with these two locations (Figure 2); indeed, for *P. charcoti*, every individual was estimated to possess a somewhat similar proportion of each of the three model clusters (Table S14). STRUCTURE analysis also showed no distinct grouping of *A. polymorpha* individuals between the Peninsula region (Q_1_ **=* *0.15, Q_2_ **=* *0.22, Q_3_ **=* *0.20, Q_5_ **=* *0.18) and Elephant Island (Q_1_ **=* *0.14, Q_2_ **=* *0.23, Q_3_ **=* *0.20, Q_5_ **=* *0.19) (Figure 2) and the level of genetic differentiation between these localities was low (*F*_ST_ **=* *0.006) but significantly different from 0 (Table 3). By contrast, STRUCTURE analysis of *P. turqueti* revealed structuring around the Peninsula, Elephant Island, and Signy Islands (Figure 2). Moreover, values of *F*_ST_ were highest (and significantly different from 0) between Signy and Elephant Island (0.055) followed by the comparisons between Signy and Livingstone Island (0.051), and Signy and King George Island (0.048) (Table 3). Thus, the model clusters identified by STRUCTURE for *P. turqueti* showed some geographic structure at a regional scale, corresponding to (3) Elephant Island (Q_2_ **=* *0.49) and to a lesser extent Signy (Q_2_ **=* *0.18) and the Peninsula (Q_2_ **=* *0.14), (4) the Peninsula (Q_3_ **=* *0.56) and to a lesser extent Signy (Q_3_ **=* *0.29) and (6) Signy (Q_6_ **=* *0.35) and to a lesser extent the Peninsula (Q_6_ **=* *0.17) and Elephant Island (Q_6_ **=* *0.12). Regional genetic differences among *P. turqueti* samples were identified using BARRIER (Manni et al., 2004), with the second barrier occurring between Shag Rocks and South Georgia; subsequent barriers were identified between Signy and the Peninsula, and finally among the samples from the Peninsula itself (Supporting Information).

The statistical power of the *P. turqueti* and *A. polymorpha* datasets was comparable and was able to detect a true *F*_ST_ of 0.0025 or more with a probability of 100% (Supporting Information). The *P. charcoti* dataset has a high probability (0.980 for Chi^2^ and 0.727 for Fisher's exact test) of detecting true values of *F*_ST_ as low as 0.005 (Supporting Information).

### 3.3 Directional migration

There was an apparent directional bias to the relative migration in *P. turqueti*, with significantly higher migration rates from Signy Island into all other sample locations, particularly Elephant Island and South Georgia (relative directional migration >0.7), than in the opposite direction (Table 4); there was no evidence for significant differential migration between all remaining locations for *P. turqueti*. No significant asymmetry in relative migration rates was detected for samples of *A. polymorpha* or *P. charcoti* (Tables S17 and S18).

**Table 4:** Directional migration estimates for *Pareledone turqueti*

	Site	Source population					
		West of Peninsula	East of Peninsula	Elephant Island	Signy Island	South Georgia	Shag Rocks
Receiving population	West of Peninsula	–	0.282	0.296	**0.288**	0.209	0.219
	East of Peninsula	0.171	–	0.217	0.190	0.118	0.130
	Elephant Island	0.355	0.489	–	**0.709**	0.423	**0.465**
	Signy Island	0.108	0.106	0.118	–	0.106	0.164
	South Georgia	0.230	**0.235**	0.295	**0.665**	–	1.000
	Shag Rocks	0.179	0.156	0.209	**0.422**	0.702	–

Left column indicates where migrants travelled to; top row indicates where migrants originated from. Bold values are significant (based on 1000 bootstraps).

### 3.4 Effect of landscape upon *P. turqueti* and *A. polymorpha*

GESTE analyses indicated a strong significant association between depth and genetic differentiation among samples of *P. turqueti*. When depth was included as a single factor in GESTE, a higher probability model was obtained than when depth was excluded (Table 5). In contrast, models containing latitude, longitude, or temperature as single factors had lower probabilities than the random effects model (termed “Constant” in Table 5). Inclusion of longitude, temperature, or depth as single factors in GESTE resulted in slightly higher probability models for *A. polymorpha* than when these factors were excluded (Table 5).

**Table 5:** Potential contribution of environmental variables in explaining genetic differences among samples of the Antarctic octopus species *Pareledone turqueti* and *Adelieledone polymorpha*

Species factor(s) in model (G)	Highest probability model P(M)	P(M)	P(G1)	P(G2)	P(G1*G2)	P(G3)	P(G4)
*P. turqueti*							
Latitude (G1)	Constant	0.502	0.498				
Longitude (G1)	Constant	0.510	0.490				
Temperature (G1)	Constant	0.510	0.490				
Depth (G1)	Constant, G1	1.000	1.000				
Depth, (G1) Temp (G2)	Constant, G1*G2, G2, G1	0.240	0.381	0.371	0.240		
Latitude (G1), Longitude (G2)	Constant, G1*G2, G2, G1	0.290	0.344	0.345	0.290		
Latitude (G1), Depth (G2)	Constant, G1*G2, G2, G1	0.230	0.377	0.387	0.230		
Longitude (G1), Depth (G2)	Constant, G1*G2, G2, G1	0.227	0.370	0.389	0.227		
Latitude (G1), Longitude (G2), Depth (G3)	Constant, G3, G2	0.130	0.491	0.500		0.491	
Latitude (G1), Longitude (G2), Depth (G3), Temperature (G4)	Constant	0.068	0.489	0.485		0.493	0.492
*A. polymorpha*							
Latitude (G1)	Constant	0.508	0.492				
Longitude (G1)	Constant, G1	0.503	0.503				
Temperature (G1)	Constant, G1	0.503	0.503				
Depth (G1)	Constant, G1	0.504	0.504				
Depth, (G1) Temp (G2)	Constant, G1*G2, G2, G1	0.227	0.377	0.385	0.227		
Latitude (G1), Longitude (G2)	Constant, G1*G2, G2, G1	0.225	0.381	0.386	0.225		
Latitude (G1), Depth (G2)	Constant, G1*G2, G2, G1	0.224	0.380	0.383	0.224		
Longitude (G1), Depth (G2)	Constant, G1*G2, G2, G1	0.231	0.386	0.380	0.231		
Latitude (G1), Longitude (G2), Depth (G3)	Constant, G2	0.130	0.500	0.503		0.487	
Latitude (G1), Longitude (G2), Depth (G3), Temperature (G4)	Constant, G3, G2	0.067	0.495	0.504		0.490	0.493

Results of regression analyses performed in GESTE to examine the proportion of genetic structure explained by variation in environmental factors. Factor(s) included in a given analysis (column 1); model that best explains the data (column 2) and its probability (column 3); probability of each factor in the highest probability model (columns 4–8), including potential interactions between factors [P(G1*G2)].

When two factors were included, the highest probability models for both *P. turqueti* and *A. polymorpha* included both factors and their interaction term. In each of these two factor comparisons, the individual factors contributed more than their interactions (Table 5). For *P. turqueti* depth contributed more than temperature, latitude, or longitude. By contrast, temperature and longitude contributed more to the model of genetic structure in *A. polymorpha* than did depth in each of these two factor comparisons (Table 5).

When latitude, longitude, and depth were included as factors in a three‐factor model the highest probability model for *P. turqueti* included effects of longitude and depth, while for *A. polymorpha* the highest probability model included a contribution by longitude only. Inclusion of all four factors in models of genetic structure did not produce a higher probability model than when all four factors were not included for *P. turqueti*, but for *A. polymorpha* the highest probability four‐factor model included potential effects of longitude and depth with longitude contributing more than depth (Table 5).

**Figure 3:**
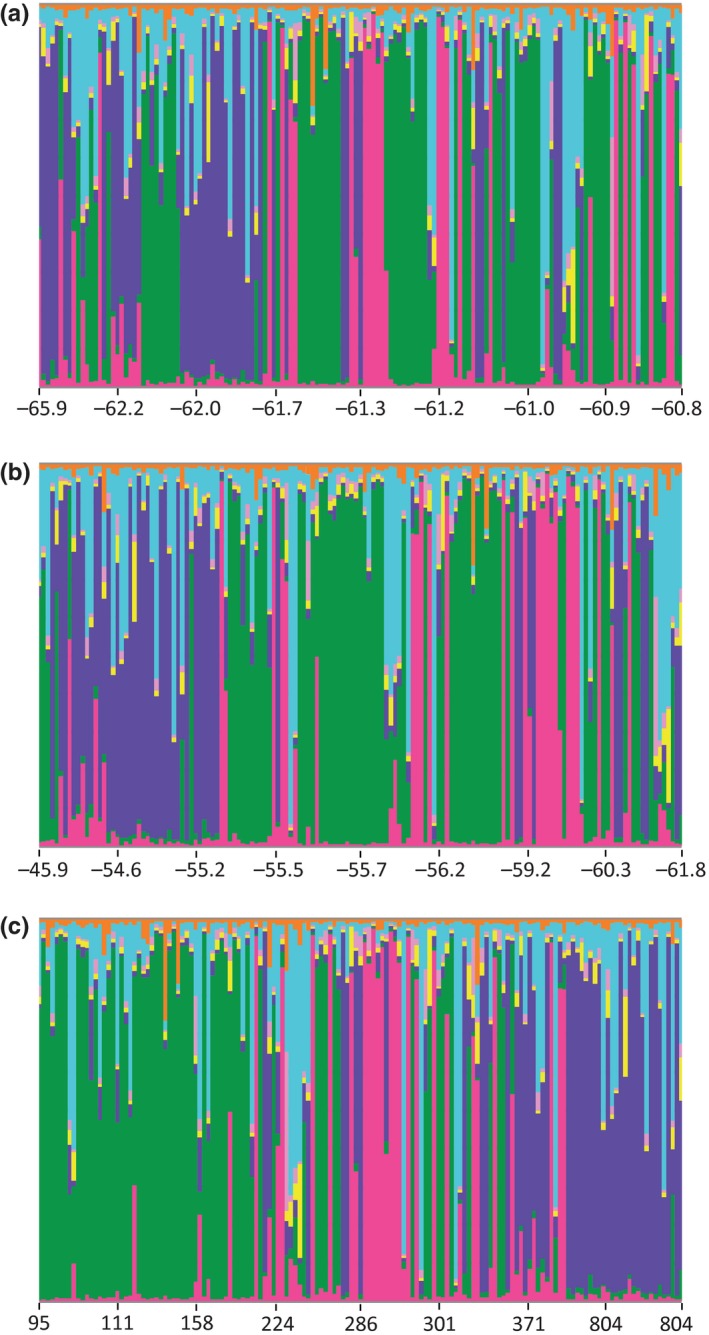
STRUCTURE assignment of individuals from Elephant Island, Signy Island, and the South Shetland Islands for *Pareledone turqueti* ordered by (a) latitude, (b) longitude, (c) depth Colors indicate percentage contribution of individuals to assigned clusters (y axis), individuals represented by each line (x axis)

STRUCTURE assignment of *P. turqueti* individuals from Elephant Island, Signy Island, and the South Shetland Islands ordered by latitude (Figure 3a), longitude (Figure 3b), and depth (Figure 3c) further indicate an association between depth and genetic differentiation among samples (Tables S19 and S20).

## 4 DISCUSSION

The present study shows that closely related octopod species with similar life history characteristics display a generally similar pattern of genetic differentiation at large geographic scales, but contrasting patterns of population genetic structure at a regional scale across the Scotia Arc region. Local environment (associated with depth) appears to play an important role in driving spatial genetic structure in *P. turqueti*, and to a lesser extent in *A. polymorpha*. Moreover, no clear spatial genetic structure was evident in *P. charcoti*, a species relatively restricted in both geographic range and depth despite the samples of this species being separated by deep water.

### 4.1 Variation in regional genetic structure

The reason(s) for the marked differences in regional spatial genetic structure between species is not clear. Although our target octopus species are closely related (Strugnell, Rogers, Prodöhl, Collins, & Allcock, 2008), we know relatively little of their basic biology. Nonetheless, the large size (>10 mm diameter) of their mature eggs (Allcock, 2005; Allcock, Hochberg, Rodhouse, & Thorpe, 2003; Allcock & Piertney, 2002; Barratt, Johnson, Collins, & Allcock, 2008) suggests that they are all direct developers (Boletzky, 1974). Populations of sedentary or sessile marine invertebrates with nonpelagic larval dispersal typically exhibit more genetic differences than species whose larvae have a long duration in the plankton (reviewed in Selkoe & Toonen, 2011). Our study area contains numerous potential barriers to dispersal to benthic invertebrates. For example, the shallowest depths between Elephant Island and the South Shetland Islands are deeper than 500 m, with the shelf areas shallower than 286 m in depth (the deepest record for *P. charcoti*) separated by about 65 km. The weak or lack of population structure between Elephant Island and the South Shetland Islands for *A. polymorpha* and *P. charcoti* is intriguing, particularly for the latter. Both *P. turqueti* and *A. polymorpha* have been caught from similarly deep habitats (1,116 m and 1,510 m, respectively) (Allcock, unpublished data in Strugnell et al., 2008) (also see Table 1), and thus, adult *A. polymorpha* might be capable of moving between these locations along the benthos. In contrast, *P. charcoti* inhabits relatively shallow depths (<286 m), with most individuals captured from ~110 m depth or less (Allcock, 2005) (Table 1). Our genetic data are counterintuitive as basic ecology indicates that populations of *P. charcoti* would exhibit the greatest genetic differentiation among sample locations. An absence of genetic differentiation among isolated populations can arise when samples have not yet attained genetic equilibrium conditions; unfortunately, samples of *P. charcoti* from the Peninsula region were difficult to obtain and the limited sample size prevents a more detailed analysis of spatial genetic structure in this region.

Greater genetic differentiation in *P. turqueti* across the Peninsula, Elephant Island, and Signy Island region, but weak genetic differences between the Peninsula and Elephant Island, indicates barriers to gene flow consistent with this species’ sedentary lifestyle and benthic young. But given their similar (and deep) bathymetric distribution why should *P. turqueti* have a more restricted dispersal than *A. polymorpha*? Preliminary studies suggest that these two genera may occupy distinct trophic niches (Daly & Rodhouse, 1994). For example, the posterior salivary glands, which contain enzymes and venoms for subduing and digesting prey are much larger in *A. polymorpha* than *P. turqueti*; these species also differ in beak morphology, with *A. polymorpha* possessing a smaller, finer beak with a rostral tip that ends in a sharper point than *P. turqueti* (Allcock et al., 2003; Daly & Rodhouse, 1994). Daly and Rodhouse (1994) speculate that the less muscular body and atypical beak of *A. polymorpha* suggests specialization for exploiting an atypical resource, possibly in the water column, whilst the robust body and more typical octopod beak of *P. turqueti* may suggest hunting of wholly benthic prey. Therefore, niche divergence driven by diet may play a role in driving genetic structure in *P. turqueti* or the lack of it in *A. polymorpha*. Unfortunately, preliminary stomach content data of these two species are not informative as octopus’ external digestion renders much of the stomach contents unidentifiable: About 50% of the stomach contents of *A. polymorpha* (*n *= 3 samples) were unidentifiable, with the remainder comprising amphipods (33%) and polychaetes (17%), whilst *P. turqueti* (*n *= 12) stomach contents also contained a high proportion of unidentifiable items (44%), amphipods (24%), polychaetes (8%) and a small proportion of other items including fish (8%), octopods (8%), and egg masses (8%); the stomach contents of *P. charcoti* (*n *= 33) comprised 90% amphipods (4% other, 6% unidentifiable) (Piatkowski, Allcock, & Vecchione, 2003). Amphipods dominate assemblages at shallow depths (<25 m) at King George Island (Jazdzewski, Teodorczyk, Sicinski, & Kontek, 1991) and in the Ross Sea amphipod abundance declines markedly with increasing depth (Lörz, Kaiser, & Bowden, 2012). Therefore, it is possible that *P. charcoti* maybe be constrained to shallow waters due to the distribution of its preferred prey.

Although neither the adults nor juveniles of *P. charcoti* (or *P. turqueti*) are expected to move between Elephant Island and the South Shetland Islands, rafting on macroalgae (e.g., Leese, Agrawal, & Held, 2010) or sea ice (see Gutt, 2001 for a review) may facilitate gene flow in this species as it does in other benthic Southern Ocean species. Anchor ice may also dislodge eggs attached to a benthic substrate that could then be moved by currents. Also, it is possible that populations of *P. charcoti* at Elephant Island and South Shetland Islands are isolated, with no contemporary dispersal, but that this species has not reached genetic equilibrium.

Low genetic diversity implies a low population size. Indeed, the genetically least diverse species *P. charcoti* has a limited distribution, occurring around the South Shetland Islands and off Graham Land (Allcock, 2005), compared with the most diverse species *P. turqueti* which has a circumpolar distribution (Strugnell et al., 2012) and also *A. polymorpha* (intermediate level of genetic diversity) which is distributed across the entire Scotia Arc (with the exception of Shag Rocks). These interspecific differences in genetic diversity are reflected in the mitochondrial diversity, whereby just eight COI haplotypes (differing by at most by five substitutions) are known for *P. charcoti*, (Allcock et al., 2011), compared with some 35 COI haplotypes reported for *P. turqueti* (Strugnell et al., 2012). In addition, *P. charcoti* and another closely related species, *P. aequipapillae* Allcock, 2005 are estimated to have diverged relatively recently (~1 mya) (Strugnell et al., 2008), whereas *P. turqueti* is thought to be an older species, (the two main lineages within *P. turqueti* were estimated to have diverged ~4 mya [95% highest posterior density 1.9–7.1 mya]) (Strugnell et al., 2012). One reason for a relatively narrow geographic and depth distribution and low levels of genetic diversity is that *P. charcoti* is a comparatively new species, supporting the idea that populations of this species are in genetic nonequilibrium (as discussed above). Alternatively, this species may have gone through a genetic bottleneck, potentially impacted by loss of habitat during the last glacial maximum (Thatje, Hillenbrand, & Larter, 2005).

### 4.2 Structure between South Georgia and the South Shetland Islands

Octopus species show a more similar pattern of spatial genetic structure at a larger geographic scale, with genetic differentiation apparent between the islands of South Georgia and continental Antarctica for *A. polymorpha* and *P. turqueti* (*P. charcoti*'s range does not extend to South Georgia). A barrier to gene flow and concomitant genetic differences between South Georgia and continental Antarctica are a consistent feature of the Southern Ocean seascape, having been reported in other benthic invertebrates, including those with a pelagic larval phase (e.g., González‐Wevar et al., 2013; Hoffman, Clarke, Clarke, et al., 2011; Hunter & Halanych, 2008; Krabbe et al., 2010; Wilson et al., 2007). While deep water and major currents are often proposed to act as dispersal barriers between continental Antarctica and South Georgia (González‐Wevar et al., 2013; Strugnell et al., 2012), the former would be expected to be less of a barrier for species with pelagic larval phases than brooders such as octopods. The importance of life history and dominant ocean flows in shaping genetic structure across this region was recently demonstrated by Young et al. (2015) who found that differences in the length of the juvenile planktonic stage and the variability in ocean flows explained differences in genetic structuring between two species of teleost.

Moreover, relatively low expected heterozygosity for *P. turqueti* and *A. polymorpha* at South Georgia (and at Shag Rocks for *P. turqueti*) is consistent with these being small island habitats (Frankham, 1997) and at the range margin of both species (e.g., Arnaud‐Haond et al., 2006; Lind, Evans, Taylor, & Jerry, 2007). Despite this low genetic diversity, the estimated prevailing direction of migration was from Signy outwards, to South Georgia and also from Signy to Elephant Island (directional relative migration >0.7), for *P. turqueti*. This asymmetric migration pattern from Signy to South Georgia is in accordance with the prevailing dispersal direction indicated from drifter buoys (Matschiner, Hanel, & Salzburger, 2009) and the pattern of dispersal detected for teleost fish (Young et al., 2015). Conversely, the high level of asymmetric migration from Signy Island to Elephant Island estimated for *P. turqueti* is surprising, as the predominantly northward flow of Weddell Sea water between Elephant Island and the South Orkney Islands has been suggested to act as a barrier to gene flow between these regions (Thompson, Heywood, Thrope, Renner, & Trasvina, 2009; Thompson & Youngs, 2013; Young et al., 2015). Clearly these genetic data need examining further in other species. However, if confirmed, asymmetric migration from Signy Island to many other locations indicates this area acts an important source population. This feature provides additional support for the importance of the Marine Protected Area that was established in this region in 2009 (CCAMLR Conservation Measure 91‐03) due to its role as a key predator foraging area.

### 4.3 Population structuring by depth across Peninsula, Elephant Island, and Signy Island

An apparent effect of depth upon genetic structure of *P. turqueti* populations from the Scotia Arc is novel for Southern Ocean species. Previous attempts to quantify the effect of bathymetry on genetic divergence in Southern Ocean taxa have been hampered by limited sample sizes and the common discovery of cryptic species (further impacting sample sizes) (Baird, Miller, & Stark, 2011). By contrast, genetic “isolation by depth” has been reported for deep‐sea species, such as the giant amphipod, *Eurythenes gryllus* (France & Kocher, 1996), the bivalve, *Deminucula atacellana* (Zardus, Etter, Chase, Rex, & Boyle, 2006), the octocoral, *Callogorgia delta* (Quattrini, Baums, Shank, Morrison, & Cordes, 2015) as well as in teleosts inhabiting more shallow areas (Shum, Pampoulie, Sacchi, & Mariani, 2014). In Southern Ocean taxa, some evidence for genetic differentiation driven by depth is derived from divergent populations (from ~630 m and 123–540 m depth) of the giant Antarctic isopod, *Glyptonotus antarcticus*, but small (*n *= 2) sample sizes from the deep population prevent a robust analysis (Held & Wägele, 2005). Conversely, no evidence for genetic structuring by depth was found in the Southern Ocean nudibranch *Doris kerguelenensis;* however, a high incidence of cryptic species may have reduced the power to detect any intraspecific patterns of population structure (Wilson et al., 2009).

The effect of depth upon *P. turqueti* population differentiation is a likely consequence of one or more environmental or biological variables that correlate with depth, such as oxygen, type and availability of food, predation, disturbance, and/or temperature (see Gage & Tyler, 1991). Data for most of these variables are not available for the Antarctic region. However, inclusion of depth, temperature, and their interaction improved the fit of the model implying that temperature plays some role in shaping the genetic structuring evident within *P. turqueti*; indeed, depth and/or temperature may shape genetic differentiation in *A. polymorpha,* although the population sample number is limited for this species. Conversely, the relationship between population genetic structure and depth in *P. turqueti* could represent residual historic genetic structure from allopatric ice‐free refugia (potentially present during the last glacial maximum) that occupied a range of depths. However, as genetic differentiation is related to bathymetry in some deep‐sea species (France & Kocher, 1996; Schüller, 2011; Zardus et al., 2006), it seems likely that one or more (as yet unidentified) environmental factors (i.e. other than glaciation) can drive population structure in benthic marine invertebrates. An intriguing possibility is that this type of environmentally driven genetic structure in Southern Ocean species promotes allopatric speciation, whereby genetically different populations experience yet further divergence in refugia during glacial cycles.

We could not examine the effect of depth on *P. charcoti* as significant population differentiation was not detected. A lack of population divergence in *P. charcoti*, a species with a restricted geographic distribution and narrow bathymetric range, might reflect fewer opportunities for diversifying selection. Nonetheless, depth may have played an important role in the evolution of *P. charcoti* as a species, as each of the seven sympatric papillated *Pareledone* species from the Antarctic Peninsula region (all of which were previously ascribed to a single species) occupied in some cases distinct and relatively narrow depth distributions (Allcock, 2005). Therefore, it is possible that ecological speciation through niche divergence may have occurred within this clade of papillated *Pareledone* species, and this may have been at least partially driven by a similar process of genetic structuring across depth as is observed in populations of *P. turqueti* in the present study.

In conclusion, these data highlight marked differences in genetic structure among three closely related octopus species. The outcome of no detectable spatial structure in the species with the most restricted geographic and bathymetric range highlights a lack of knowledge about local scale population structuring in Antarctic taxa. Moreover, these data highlight complexity of processes that impact population structure in a limited region in species that are apparently biologically similar. This highlights a potential difficulty in managing benthic Antarctic species from data on just a single “typical species.” In addition, local associations between depth and genetic variation in *P. turqueti* may be suggestive of a previously unrecognized driver of ecological speciation in Southern Ocean benthos.

## CONFLICT OF INTEREST

None declared.

## AUTHORSHIP

JMS performed the research, JMS and PCW analyzed the data and wrote the manuscript, JMS, PCW, and ALA designed the research.
