# Supplementary material for: Closely related octopus species show different spatial genetic structures in response to the Antarctic seascape
Source: Ecol Evol. 2017 Sep 5;7(19):8087–99. doi: 10.1002/ece3.3327 (PMC5632630; doi:10.1002/ece3.3327)
Supplement: Supplementary file 1 [file ECE3-7-8087-s001.docx]

**Supporting Information**

**S1**

**Table 1.** Probability of deviating from expected Hardy-Weinberg equilibrium (HWE) conditions for large samples of the Antarctic benthic octopus *P. charcoti*. Values that remain significant (*P*<0.05) after a sequential Bonferroni correction (*k*=10 loci) (Rice 1989) are highlighted bold.

| Locus | Elephant Island | Peninsula |
| --- | --- | --- |
| PC_B01_B06 | 0.1367 | 0.2521 |
| PC_B03_B08 | **0.0032** | 1.0000 |
| PC_C05_C10 | **0.0000** | 0.1289 |
| PC_G01_G06 | **0.0000** | 1.0000 |
| PC_D02_D07 | 0.1444 | 0.1289 |
| PC_G03_G08 | **0.0000** | 0.4870 |
| PC_H02_H07 | 0.0137 | 0.8604 |
| PT_E04_E09 | **0.0000** | 1.0000 |
| PT_F03_F08 | **0.0000** | 0.1711 |
| PT_F02_F07 | 0.1655 | 0.7782 |

**Table 2.** Probability of deviating from expected Hardy-Weinberg equilibrium (HWE) conditions for large samples of the Antarctic benthic octopus *A. polymorpha*. Values that remain significant (*P*<0.05) after a sequential Bonferroni correction (*k*=9 loci) (Rice 1989) are highlighted bold.

| Locus | Elephant Island | Signy Island | Peninsula | South Georgia |
| --- | --- | --- | --- | --- |
| AP_H02 | **0.0010** | 1.0000 | 0.0989 | 0.1953 |
| AP_B09 | 0.0476 | 1.0000 | 0.2814 | **0.0000** |
| AP_E10 | **0.0000** | - | 0.1766 | **0.0032** |
| AP_A09 | 0.0459 | - | **0.0000** | 0.0748 |
| AP_H09 | 0.2545 | - | 0.2517 | 0.2307 |
| AP_B02 | **0.0000** | 1.0000 | 0.0655 | 0.1936 |
| AP_H10 | **0.0000** | 1.0000 | 0.1249 | 0.6851 |
| AP_G05 | **0.0013** | 1.0000 | 0.0212 | **0.0000** |
| AP_D04 | 0.7899 | 0.3322 | 0.0820 | 0.1807 |

**S2**

**Table 3.** Tests for linkage disequilibrium for *P. charcoti.* P-value for each locus pair across all populations (Fisher’s method)

| Locus | Locus | Chi2 | df | P-Value |
| --- | --- | --- | --- | --- |
| PC_B01_B06 | PC_B03_B08 | 1.035331 | 2 | 0.59591 |
| PC_B01_B06 | PC_C05_C10 | 6.762637 | 4 | 0.148976 |
| PC_B03_B08 | PC_C05_C10 | 2.032664 | 2 | 0.36192 |
| PC_B01_B06 | PC_G01_G06 | 1.697264 | 4 | 0.791215 |
| PC_B03_B08 | PC_G01_G06 | 0.845629 | 2 | 0.6552 |
| PC_C05_C10 | PC_G01_G06 | 6.981899 | 4 | 0.136848 |
| PC_B01_B06 | PC_D02_D07 | 0.039222 | 2 | 0.98058 |
| PC_B03_B08 | PC_D02_D07 | 2.371546 | 2 | 0.30551 |
| PC_C05_C10 | PC_D02_D07 | 1.129262 | 2 | 0.56857 |
| PC_G01_G06 | PC_D02_D07 | 9.878491 | 2 | **0.00716** |
| PC_B01_B06 | PC_G03_G08 | 4.68869 | 2 | 0.09591 |
| PC_B03_B08 | PC_G03_G08 | 0 | 2 | 1 |
| PC_C05_C10 | PC_G03_G08 | 1.745026 | 2 | 0.4179 |
| PC_G01_G06 | PC_G03_G08 | Infinity | 2 | **Highly significant** |
| PC_D02_D07 | PC_G03_G08 | 0 | 2 | 1 |
| PC_B01_B06 | PC_H02_H07 | 0.35128 | 4 | 0.986268 |
| PC_B03_B08 | PC_H02_H07 | 1.648152 | 2 | 0.43864 |
| PC_C05_C10 | PC_H02_H07 | 12.439241 | 4 | **0.014367** |
| PC_G01_G06 | PC_H02_H07 | Infinity | 4 | **Highly significant** |
| PC_D02_D07 | PC_H02_H07 | 0.77309 | 2 | 0.6794 |
| PC_G03_G08 | PC_H02_H07 | 0.939207 | 2 | 0.62525 |
| PC_B01_B06 | PT_E04_E09 | 0.29306 | 2 | 0.8637 |
| PC_B03_B08 | PT_E04_E09 | 2.907434 | 2 | 0.2337 |
| PC_C05_C10 | PT_E04_E09 | 0 | 2 | 1 |
| PC_G01_G06 | PT_E04_E09 | 7.616227 | 2 | **0.02219** |
| PC_D02_D07 | PT_E04_E09 | 0 | 2 | 1 |
| PC_G03_G08 | PT_E04_E09 | 0.00888 | 2 | 0.99557 |
| PC_H02_H07 | PT_E04_E09 | 3.244337 | 2 | 0.19747 |
| PC_B01_B06 | PT_F03_F08 | 2.003806 | 4 | 0.735059 |
| PC_B03_B08 | PT_F03_F08 | 0.074698 | 2 | 0.96334 |
| PC_C05_C10 | PT_F03_F08 | 3.611149 | 4 | 0.46118 |
| PC_G01_G06 | PT_F03_F08 | Infinity | 4 | **Highly significant** |
| PC_D02_D07 | PT_F03_F08 | 1.737765 | 2 | 0.41942 |
| PC_G03_G08 | PT_F03_F08 | 4.581115 | 2 | 0.10121 |
| PC_H02_H07 | PT_F03_F08 | 5.964843 | 2 | 0.05067 |
| PT_E04_E09 | PT_F03_F08 | 3.564252 | 2 | 0.16828 |
| PC_B01_B06 | PT_F02_F07 | 1.872681 | 2 | 0.39206 |
| PC_B03_B08 | PT_F02_F07 | 2.143259 | 2 | 0.34245 |
| PC_C05_C10 | PT_F02_F07 | 0.052339 | 2 | 0.97417 |
| PC_G01_G06 | PT_F02_F07 | 2.984466 | 2 | 0.22487 |
| PC_D02_D07 | PT_F02_F07 | 0 | 2 | 1 |
| PC_G03_G08 | PT_F02_F07 | Infinity | 2 | **Highly significant** |
| PC_H02_H07 | PT_F02_F07 | 2.645963 | 2 | 0.26634 |
| PT_E04_E09 | PT_F02_F07 | 3.876856 | 2 | 0.14393 |
| PT_F03_F08 | PT_F02_F07 | 0.535314 | 2 | 0.76517 |

**Table 4.** Tests for linkage disequilibrium for *P charcoti*. P-value for each locus pair in each population

| Population |  | Locus | Locus | P-Value | S.E. |
| --- | --- | --- | --- | --- | --- |
| Elephant Island |  | PC_B01_B06 | PC_B03_B08 | 0.59591 | 0.048365 |
| Elephant Island |  | PC_B01_B06 | PC_C05_C10 | 0.04762 | 0.020359 |
| Elephant Island |  | PC_B03_B08 | PC_C05_C10 | 0.36192 | 0.04681 |
| Elephant Island |  | PC_B01_B06 | PC_G01_G06 | 0.428 | 0.045472 |
| Elephant Island |  | PC_B03_B08 | PC_G01_G06 | 0.6552 | 0.040863 |
| Elephant Island |  | PC_C05_C10 | PC_G01_G06 | 0.11219 | 0.026453 |
| Elephant Island |  | PC_B01_B06 | PC_D02_D07 | 0.98058 | 0.009951 |
| Elephant Island |  | PC_B03_B08 | PC_D02_D07 | 0.30551 | 0.046024 |
| Elephant Island |  | PC_C05_C10 | PC_D02_D07 | 0.56857 | 0.049121 |
| Elephant Island |  | PC_G01_G06 | PC_D02_D07 | **0.00716** | **0.00716** |
| Elephant Island |  | PC_B01_B06 | PC_G03_G08 | 0.09591 | 0.029093 |
| Elephant Island |  | PC_B03_B08 | PC_G03_G08 | 1 | 0 |
| Elephant Island |  | PC_C05_C10 | PC_G03_G08 | 0.4179 | 0.048652 |
| Elephant Island |  | PC_G01_G06 | PC_G03_G08 | **0** | **0** |
| Elephant Island |  | PC_D02_D07 | PC_G03_G08 | 1 | 0 |
| Elephant Island |  | PC_B01_B06 | PC_H02_H07 | 0.83892 | 0.035237 |
| Elephant Island |  | PC_B03_B08 | PC_H02_H07 | 0.43864 | 0.04907 |
| Elephant Island |  | PC_C05_C10 | PC_H02_H07 | **0.00199** | **0.00199** |
| Elephant Island |  | PC_G01_G06 | PC_H02_H07 | **0** | **0** |
| Elephant Island |  | PC_D02_D07 | PC_H02_H07 | 0.6794 | 0.046843 |
| Elephant Island |  | PC_G03_G08 | PC_H02_H07 | 0.62525 | 0.04839 |
| Elephant Island |  | PC_B01_B06 | PT_E04_E09 | 0.8637 | 0.033489 |
| Elephant Island |  | PC_B03_B08 | PT_E04_E09 | 0.2337 | 0.042254 |
| Elephant Island |  | PC_C05_C10 | PT_E04_E09 | 1 | 0 |
| Elephant Island |  | PC_G01_G06 | PT_E04_E09 | **0.02219** | **0.01369** |
| Elephant Island |  | PC_D02_D07 | PT_E04_E09 | 1 | 0 |
| Elephant Island |  | PC_G03_G08 | PT_E04_E09 | 0.99557 | 0.00312 |
| Elephant Island |  | PC_H02_H07 | PT_E04_E09 | 0.19747 | 0.03977 |
| Elephant Island |  | PC_B01_B06 | PT_F03_F08 | 0.36718 | 0.047131 |
| Elephant Island |  | PC_B03_B08 | PT_F03_F08 | 0.96334 | 0.017694 |
| Elephant Island |  | PC_C05_C10 | PT_F03_F08 | 0.16438 | 0.036013 |
| Elephant Island |  | PC_G01_G06 | PT_F03_F08 | **0** | **0** |
| Elephant Island |  | PC_D02_D07 | PT_F03_F08 | 0.41942 | 0.048859 |
| Elephant Island |  | PC_G03_G08 | PT_F03_F08 | 0.10121 | 0.029254 |
| Elephant Island |  | PC_H02_H07 | PT_F03_F08 | 0.05067 | 0.021899 |
| Elephant Island |  | PT_E04_E09 | PT_F03_F08 | 0.16828 | 0.036372 |
| Elephant Island |  | PC_B01_B06 | PT_F02_F07 | 0.39206 | 0.046606 |
| Elephant Island |  | PC_B03_B08 | PT_F02_F07 | 0.34245 | 0.046847 |
| Elephant Island |  | PC_C05_C10 | PT_F02_F07 | 0.97417 | 0.01499 |
| Elephant Island |  | PC_G01_G06 | PT_F02_F07 | 0.22487 | 0.031853 |
| Elephant Island |  | PC_D02_D07 | PT_F02_F07 | 1 | 0 |
| Elephant Island |  | PC_G03_G08 | PT_F02_F07 | **0** | **0** |
| Elephant Island |  | PC_H02_H07 | PT_F02_F07 | 0.26634 | 0.043019 |
| Elephant Island |  | PT_E04_E09 | PT_F02_F07 | 0.14393 | 0.034936 |
| Elephant Island |  | PT_F03_F08 | PT_F02_F07 | 0.76517 | 0.042165 |
| Peninsula |  | PC_B01_B06 | PC_B03_B08 | - | - |
| Peninsula |  | PC_B01_B06 | PC_C05_C10 | 0.71404 | 0.014891 |
| Peninsula |  | PC_B03_B08 | PC_C05_C10 | - | - |
| Peninsula |  | PC_B01_B06 | PC_G01_G06 | 1 | 0 |
| Peninsula |  | PC_B03_B08 | PC_G01_G06 | - | - |
| Peninsula |  | PC_C05_C10 | PC_G01_G06 | 0.27161 | 0.005828 |
| Peninsula |  | PC_B01_B06 | PC_D02_D07 | - | - |
| Peninsula |  | PC_B03_B08 | PC_D02_D07 | - | - |
| Peninsula |  | PC_C05_C10 | PC_D02_D07 | - | - |
| Peninsula |  | PC_G01_G06 | PC_D02_D07 | - | - |
| Peninsula |  | PC_B01_B06 | PC_G03_G08 | - | - |
| Peninsula |  | PC_B03_B08 | PC_G03_G08 | - | - |
| Peninsula |  | PC_C05_C10 | PC_G03_G08 | - | - |
| Peninsula |  | PC_G01_G06 | PC_G03_G08 | - | - |
| Peninsula |  | PC_D02_D07 | PC_G03_G08 | - | - |
| Peninsula |  | PC_B01_B06 | PC_H02_H07 | 1 | 0 |
| Peninsula |  | PC_B03_B08 | PC_H02_H07 | - | - |
| Peninsula |  | PC_C05_C10 | PC_H02_H07 | 1 | 0 |
| Peninsula |  | PC_G01_G06 | PC_H02_H07 | 0.71313 | 0.006026 |
| Peninsula |  | PC_D02_D07 | PC_H02_H07 | - | - |
| Peninsula |  | PC_G03_G08 | PC_H02_H07 | - | - |
| Peninsula |  | PC_B01_B06 | PT_E04_E09 | - | - |
| Peninsula |  | PC_B03_B08 | PT_E04_E09 | - | - |
| Peninsula |  | PC_C05_C10 | PT_E04_E09 | - | - |
| Peninsula |  | PC_G01_G06 | PT_E04_E09 | - | - |
| Peninsula |  | PC_D02_D07 | PT_E04_E09 | - | - |
| Peninsula |  | PC_G03_G08 | PT_E04_E09 | - | - |
| Peninsula |  | PC_H02_H07 | PT_E04_E09 | - | - |
| Peninsula |  | PC_B01_B06 | PT_F03_F08 | 1 | 0 |
| Peninsula |  | PC_B03_B08 | PT_F03_F08 | - | - |
| Peninsula |  | PC_C05_C10 | PT_F03_F08 | 1 | 0 |
| Peninsula |  | PC_G01_G06 | PT_F03_F08 | 0.79492 | 0.006104 |
| Peninsula |  | PC_D02_D07 | PT_F03_F08 | - | - |
| Peninsula |  | PC_G03_G08 | PT_F03_F08 | - | - |
| Peninsula |  | PC_H02_H07 | PT_F03_F08 | - | - |
| Peninsula |  | PT_E04_E09 | PT_F03_F08 | - | - |
| Peninsula |  | PC_B01_B06 | PT_F02_F07 | - | - |
| Peninsula |  | PC_B03_B08 | PT_F02_F07 | - | - |
| Peninsula |  | PC_C05_C10 | PT_F02_F07 | - | - |
| Peninsula |  | PC_G01_G06 | PT_F02_F07 | - | - |
| Peninsula |  | PC_D02_D07 | PT_F02_F07 | - | - |
| Peninsula |  | PC_G03_G08 | PT_F02_F07 | - | - |
| Peninsula |  | PC_H02_H07 | PT_F02_F07 | - | - |
| Peninsula |  | PT_E04_E09 | PT_F02_F07 | - | - |
| Peninsula |  | PT_F03_F08 | PT_F02_F07 | - | - |

**Table 5.** Tests for linkage disequilibrium for *A. polymorpha*. P-value for each locus pair across all populations (Fisher’s method)

| Locus | Locus | Chi2 | df | P-Value |  |
| --- | --- | --- | --- | --- | --- |
| AP_H02 | AP_B09 | 5.39992 | 6 | 0.493634 |  |
| AP_H02 | AP_E10 | 3.41127 | 6 | 0.755735 |  |
| AP_B09 | AP_E10 | 1.972956 | 6 | 0.922169 |  |
| AP_H02 | AP_A09 | 8.342848 | 6 | 0.214046 |  |
| AP_B09 | AP_A09 | 9.284791 | 6 | 0.158184 |  |
| AP_E10 | AP_A09 | Infinity | 6 | **Highly significant** |  |
| AP_H02 | AP_H09 | 8.031918 | 6 | 0.235774 |  |
| AP_B09 | AP_H09 | 2.361038 | 6 | 0.883684 |  |
| AP_E10 | AP_H09 | 11.211775 | 6 | 0.082048 |  |
| AP_A09 | AP_H09 | 3.206516 | 6 | 0.782516 |  |
| AP_H02 | AP_B02 | Infinity | 6 | **Highly significant** |  |
| AP_B09 | AP_B02 | Infinity | 6 | **Highly significant** |  |
| AP_E10 | AP_B02 | 4.92942 | 6 | 0.552897 |  |
| AP_A09 | AP_B02 | 8.105484 | 6 | 0.230477 |  |
| AP_H09 | AP_B02 | 3.645424 | 6 | 0.724532 |  |
| AP_H02 | AP_H10 | Infinity | 6 | **Highly significant** |  |
| AP_B09 | AP_H10 | 8.792589 | 6 | 0.185583 |  |
| AP_E10 | AP_H10 | 7.363376 | 6 | 0.288545 |  |
| AP_A09 | AP_H10 | 10.693631 | 6 | 0.098319 |  |
| AP_H09 | AP_H10 | 2.982958 | 6 | 0.810983 |  |
| AP_B02 | AP_H10 | 9.865039 | 6 | 0.130452 |  |
| AP_H02 | AP_G05 | 13.273545 | 6 | 0.038892 |  |
| AP_B09 | AP_G05 | 8.797557 | 6 | 0.185288 |  |
| AP_E10 | AP_G05 | 21.099791 | 6 | **0.00176** |  |
| AP_A09 | AP_G05 | 6.332473 | 6 | 0.386995 |  |
| AP_H09 | AP_G05 | 9.493543 | 6 | 0.147665 |  |
| AP_B02 | AP_G05 | 2.747769 | 6 | 0.839773 |  |
| AP_H10 | AP_G05 | 10.047435 | 6 | 0.122669 |  |
| AP_H02 | AP_D04 | 6.867131 | 6 | 0.33331 |  |
| AP_B09 | AP_D04 | 7.733106 | 6 | 0.258316 |  |
| AP_E10 | AP_D04 | 7.354275 | 6 | 0.289323 |  |
| AP_A09 | AP_D04 | 1.984395 | 6 | 0.921128 |  |
| AP_H09 | AP_D04 | 4.720356 | 6 | 0.580147 |  |
| AP_B02 | AP_D04 | 2.936878 | 6 | 0.816727 |  |
| AP_H10 | AP_D04 | 11.185026 | 6 | 0.082824 |  |
| AP_G05 | AP_D04 | 7.726974 | 6 | 0.258796 |  |

**Table 6.** Tests for linkage disequilibrium for *A. polymorpha*. P-value for each locus pair in each population

| Population |  | Locus | Locus | P-Value | S.E. |
| --- | --- | --- | --- | --- | --- |
| Elephant Island |  | AP_H02 | AP_B09 | 0.23397 | 0.040809 |
| Elephant Island |  | AP_H02 | AP_E10 | 0.40179 | 0.046484 |
| Elephant Island |  | AP_B09 | AP_E10 | 0.59554 | 0.04768 |
| Elephant Island |  | AP_H02 | AP_A09 | 0.43601 | 0.044575 |
| Elephant Island |  | AP_B09 | AP_A09 | 0.0498 | 0.020548 |
| Elephant Island |  | AP_E10 | AP_A09 | **0** | **0** |
| Elephant Island |  | AP_H02 | AP_H09 | 0.14221 | 0.033668 |
| Elephant Island |  | AP_B09 | AP_H09 | 0.66911 | 0.046302 |
| Elephant Island |  | AP_E10 | AP_H09 | 0.10127 | 0.028593 |
| Elephant Island |  | AP_A09 | AP_H09 | 0.49971 | 0.046009 |
| Elephant Island |  | AP_H02 | AP_B02 | 0.74384 | 0.03865 |
| Elephant Island |  | AP_B09 | AP_B02 | 0.09177 | 0.026124 |
| Elephant Island |  | AP_E10 | AP_B02 | 0.30364 | 0.040305 |
| Elephant Island |  | AP_A09 | AP_B02 | 0.0502 | 0.0163 |
| Elephant Island |  | AP_H09 | AP_B02 | 0.41977 | 0.046238 |
| Elephant Island |  | AP_H02 | AP_H10 | **0** | **0** |
| Elephant Island |  | AP_B09 | AP_H10 | 0.07968 | 0.024132 |
| Elephant Island |  | AP_E10 | AP_H10 | 0.36778 | 0.043367 |
| Elephant Island |  | AP_A09 | AP_H10 | 0.11402 | 0.025244 |
| Elephant Island |  | AP_H09 | AP_H10 | 0.68362 | 0.044187 |
| Elephant Island |  | AP_B02 | AP_H10 | 0.03297 | 0.014762 |
| Elephant Island |  | AP_H02 | AP_G05 | 0.97869 | 0.010778 |
| Elephant Island |  | AP_B09 | AP_G05 | 0.2149 | 0.040975 |
| Elephant Island |  | AP_E10 | AP_G05 | **0.01033** | **0.005154** |
| Elephant Island |  | AP_A09 | AP_G05 | 0.55289 | 0.044818 |
| Elephant Island |  | AP_H09 | AP_G05 | 0.03636 | 0.016703 |
| Elephant Island |  | AP_B02 | AP_G05 | 0.32637 | 0.038342 |
| Elephant Island |  | AP_H10 | AP_G05 | **0.0289** | **0.009409** |
| Elephant Island |  | AP_H02 | AP_D04 | 0.7254 | 0.043108 |
| Elephant Island |  | AP_B09 | AP_D04 | 0.61128 | 0.047982 |
| Elephant Island |  | AP_E10 | AP_D04 | 0.6518 | 0.04582 |
| Elephant Island |  | AP_A09 | AP_D04 | 0.87059 | 0.032491 |
| Elephant Island |  | AP_H09 | AP_D04 | 0.95219 | 0.021012 |
| Elephant Island |  | AP_B02 | AP_D04 | 0.41682 | 0.043739 |
| Elephant Island |  | AP_H10 | AP_D04 | 0.08506 | 0.024462 |
| Elephant Island |  | AP_G05 | AP_D04 | 0.71997 | 0.044379 |
| Peninsula |  | AP_H02 | AP_B09 | 0.36779 | 0.043785 |
| Peninsula |  | AP_H02 | AP_E10 | 0.83836 | 0.03087 |
| Peninsula |  | AP_B09 | AP_E10 | 0.81824 | 0.031473 |
| Peninsula |  | AP_H02 | AP_A09 | 0.63377 | 0.043984 |
| Peninsula |  | AP_B09 | AP_A09 | 0.53657 | 0.037938 |
| Peninsula |  | AP_E10 | AP_A09 | 0.13837 | 0.02998 |
| Peninsula |  | AP_H02 | AP_H09 | 0.78778 | 0.037468 |
| Peninsula |  | AP_B09 | AP_H09 | 0.86006 | 0.033923 |
| Peninsula |  | AP_E10 | AP_H09 | 0.33429 | 0.043973 |
| Peninsula |  | AP_A09 | AP_H09 | 0.52792 | 0.046381 |
| Peninsula |  | AP_H02 | AP_B02 | **0** | **0** |
| Peninsula |  | AP_B09 | AP_B02 | **0.01929** | **0.00779** |
| Peninsula |  | AP_E10 | AP_B02 | 0.58012 | 0.035621 |
| Peninsula |  | AP_A09 | AP_B02 | 0.5609 | 0.036319 |
| Peninsula |  | AP_H09 | AP_B02 | 0.45698 | 0.041533 |
| Peninsula |  | AP_H02 | AP_H10 | 0.17709 | 0.034183 |
| Peninsula |  | AP_B09 | AP_H10 | 0.2675 | 0.038174 |
| Peninsula |  | AP_E10 | AP_H10 | 0.97683 | 0.012838 |
| Peninsula |  | AP_A09 | AP_H10 | 0.10191 | 0.023748 |
| Peninsula |  | AP_H09 | AP_H10 | 0.47506 | 0.047287 |
| Peninsula |  | AP_B02 | AP_H10 | 0.32222 | 0.034118 |
| Peninsula |  | AP_H02 | AP_G05 | **0.0122** | **0.008161** |
| Peninsula |  | AP_B09 | AP_G05 | 0.54995 | 0.04654 |
| Peninsula |  | AP_E10 | AP_G05 | 0.0624 | 0.020538 |
| Peninsula |  | AP_A09 | AP_G05 | 0.96614 | 0.013051 |
| Peninsula |  | AP_H09 | AP_G05 | 0.82324 | 0.036517 |
| Peninsula |  | AP_B02 | AP_G05 | 0.87872 | 0.026057 |
| Peninsula |  | AP_H10 | AP_G05 | 0.37203 | 0.043591 |
| Peninsula |  | AP_H02 | AP_D04 | 0.46874 | 0.047063 |
| Peninsula |  | AP_B09 | AP_D04 | 0.04442 | 0.018456 |
| Peninsula |  | AP_E10 | AP_D04 | 0.93876 | 0.021166 |
| Peninsula |  | AP_A09 | AP_D04 | 0.69876 | 0.039309 |
| Peninsula |  | AP_H09 | AP_D04 | 0.2659 | 0.043718 |
| Peninsula |  | AP_B02 | AP_D04 | 0.55248 | 0.036651 |
| Peninsula |  | AP_H10 | AP_D04 | 0.15138 | 0.031858 |
| Peninsula |  | AP_G05 | AP_D04 | 0.58614 | 0.048539 |
| South Georgia |  | AP_H02 | AP_B09 | 0.78102 | 0.03476 |
| South Georgia |  | AP_H02 | AP_E10 | 0.53929 | 0.046348 |
| South Georgia |  | AP_B09 | AP_E10 | 0.76522 | 0.03638 |
| South Georgia |  | AP_H02 | AP_A09 | 0.05584 | 0.019139 |
| South Georgia |  | AP_B09 | AP_A09 | 0.36056 | 0.039574 |
| South Georgia |  | AP_E10 | AP_A09 | 0.06391 | 0.018594 |
| South Georgia |  | AP_H02 | AP_H09 | 0.1609 | 0.029978 |
| South Georgia |  | AP_B09 | AP_H09 | 0.53368 | 0.046866 |
| South Georgia |  | AP_E10 | AP_H09 | 0.10859 | 0.022508 |
| South Georgia |  | AP_A09 | AP_H09 | 0.76283 | 0.03177 |
| South Georgia |  | AP_H02 | AP_B02 | 0.07239 | 0.02204 |
| South Georgia |  | AP_B09 | AP_B02 | **0** | **0** |
| South Georgia |  | AP_E10 | AP_B02 | 0.48274 | 0.046163 |
| South Georgia |  | AP_A09 | AP_B02 | 0.61706 | 0.042459 |
| South Georgia |  | AP_H09 | AP_B02 | 0.84236 | 0.029393 |
| South Georgia |  | AP_H02 | AP_H10 | **0** | **0** |
| South Georgia |  | AP_B09 | AP_H10 | 0.57815 | 0.037178 |
| South Georgia |  | AP_E10 | AP_H10 | 0.07009 | 0.01829 |
| South Georgia |  | AP_A09 | AP_H10 | 0.40993 | 0.035192 |
| South Georgia |  | AP_H09 | AP_H10 | 0.69294 | 0.035106 |
| South Georgia |  | AP_B02 | AP_H10 | 0.67852 | 0.032557 |
| South Georgia |  | AP_H02 | AP_G05 | 0.10982 | 0.028816 |
| South Georgia |  | AP_B09 | AP_G05 | 0.10401 | 0.028665 |
| South Georgia |  | AP_E10 | AP_G05 | 0.04064 | 0.016577 |
| South Georgia |  | AP_A09 | AP_G05 | 0.07893 | 0.023283 |
| South Georgia |  | AP_H09 | AP_G05 | 0.28997 | 0.041182 |
| South Georgia |  | AP_B02 | AP_G05 | 0.88261 | 0.030918 |
| South Georgia |  | AP_H10 | AP_G05 | 0.612 | 0.037226 |
| South Georgia |  | AP_H02 | AP_D04 | 0.09491 | 0.027643 |
| South Georgia |  | AP_B09 | AP_D04 | 0.77083 | 0.040779 |
| South Georgia |  | AP_E10 | AP_D04 | 0.04134 | 0.017392 |
| South Georgia |  | AP_A09 | AP_D04 | 0.60947 | 0.044997 |
| South Georgia |  | AP_H09 | AP_D04 | 0.37286 | 0.046714 |
| South Georgia |  | AP_B02 | AP_D04 | 1 | 0 |
| South Georgia |  | AP_H10 | AP_D04 | 0.28934 | 0.037928 |
| South Georgia |  | AP_G05 | AP_D04 | 0.04975 | 0.019946 |

**Table 7.** Descriptive statistics across all populations of *Adelieledone polymorpha* (nulls not corrected)

| **Population** | ***N*** | ***N_A_*** | ***N_PA_*** | ***A_R_*** | ***H_O_*** | ***H_E_*** |
| --- | --- | --- | --- | --- | --- | --- |
| Elephant Island | 108 | 18.78 | 6.11 | 1.64 | 0.428 | 0.568 |
| Peninsula | 68 | 14.56 | 3.44 | 1.62 | 0.400 | 0.553 |
| South Georgia | 111 | 18.44 | 7.44 | 1.67 | 0.382 | 0.533 |

*N*, number of individuals per population; *N_A_*, average number of alleles across all loci per population; *N_PA_*, average number of private alleles across all loci per population; *A_R_*, average allelic richness across all loci per population (standardized to 1 individual); *H_O_*, observed level of heterozygosity; *H_E_*, expected level of heterozygosity.

**Table 8.** Descriptive statistics across all populations of *Pareledone charcoti* (nulls not corrected)

| **Population** | ***N*** | ***N_A_*** | ***N_PA_*** | ***A_R_*** | ***H_O_*** | ***H_E_*** |
| --- | --- | --- | --- | --- | --- | --- |
| Elephant Island | 350 | 36.5 | 29.3 | 5.439 | 0.272 | 0.36 |
| Peninsula | 11 | 7.5 | 0.4 | 5.595s | 0.289 | 0.382 |

*N*, number of individuals per population; *N_A_*, average number of alleles across all loci per population; *N_PA_*, average number of private alleles across all loci per population; *A_R_*, average allelic richness across all loci per population (standardized to 5 individuals); *H_O_*, observed level of heterozygosity; H_E_, expected level of heterozygosity.

**Table 9.** Descriptive statistics across all populations of *Pareledone turqueti* (nulls not corrected, but with SG and SR cut back in number of individuals to allow Fstat to run)

| **Population** | ***N*** | ***N_A_*** | ***N_PA_*** | ***A_R_*** | ***H_O_*** | ***H_E_*** |
| --- | --- | --- | --- | --- | --- | --- |
| Elephant Island | 93 | 34.3 | 7 | 10.0 | 0.7602 | 0.7429 |
| Signy | 9 | 8.1 | 0.8 | 8.1 | 0.7444 | 0.7379 |
| South Georgia | 182 | 20.5 | 7.6 | 9.103 | 0.6841 | 0.6939 |
| Shag Rocks | 125 | 19.6 | 5.6 | 8.528 | 0.744 | 0.7297 |
| Peninsula (EAP + WAP) | 46 | 25 | 3.3 | 10.152 | 0.7326 | 0.7333 |

*N*, number of individuals per population; *N_A_*, average number of alleles across all loci per population; *N_PA_*, average number of private alleles across all loci per population; *A_R_*, average allelic richness across all loci per population (standardized to 9 individuals); *H_O_*, observed level of heterozygosity; *H_E_*, expected level of heterozygosity.

**Table 10.** F-statistics across all populations of *A. polymorpha* (nulls not corrected).

|  | Elephant Island | Signy | Peninsula | South Georgia |
| --- | --- | --- | --- | --- |
| Elephant Island | - | NA | NS | * |
| Signy | 0.0214 | - | NA | NA |
| Peninsula | 0.0021 | 0.0143 | - | * |
| South Georgia | 0.0342 | 0.0489 | 0.0447 | - |

Significance after Bonferroni correction above diagonal. Fst below the diagonal.

P values obtained after: 120 permutations. Indicative adjusted nominal level (5%) for multiple comparisons is 0.008333.

**Table 11.** F-statistics across all populations of *P. charcoti* (nulls not corrected).

|  | Elephant Island | Peninsula |
| --- | --- | --- |
| Elephant Island | - | NA |
| Peninsula | 0.0089 | - |

Significance after Bonferroni correction above diagonal. Fst below the diagonal.

P values obtained after: 20 permutations. Indicative adjusted nominal level (5%) for multiple comparisons is 0.05.

**Table 12.** F-statistics across all populations of *P. turqueti* (nulls not corrected).

|  | Peninsula | Elephant Island | Signy Island | South Georgia | Shag Rocks |
| --- | --- | --- | --- | --- | --- |
| Peninsula | - | * (p = 0.005) | NS (p = 0.025) | * (p = 0.005) | * (p = 0.005) |
| Elephant Island | 0.0286 | *-* | * (p = 0.005) | * (p = 0.005) | * (p = 0.005) |
| Signy Island | 0.0284 | 0.0486 | *-* | * (p = 0.005) | * (p = 0.005) |
| South Georgia | 0.0482 | 0.0375 | 0.0635 | *-* | * (p = 0.005) |
| Shag Rocks | 0.0707 | 0.0645 | 0.0840 | 0.0556 | *-* |

Significance after Bonferroni correction above diagonal. Fst below the diagonal.

P values obtained after: 200 permutations. Indicative adjusted nominal level (5%) for multiple comparisons is 0.005

**Table 13.** Contribution (Q-values) of each population to assigned clusters (K = 6) using STRUCTURE for *Adelieledone polymorpha*

|  |  | Contribution to clusters | | | | | |
| --- | --- | --- | --- | --- | --- | --- | --- |
| Cluster | n | 1 | 2 | 3 | 4 | 5 | 6 |
| Elephant Island | 108 | 0.14 | 0.23 | 0.20 | 0.14 | 0.19 | 0.10 |
| Signy Island | 2 | 0.16 | 0.38 | 0.10 | 0.16 | 0.10 | 0.10 |
| Peninsula | 68 | 0.15 | 0.22 | 0.20 | 0.15 | 0.18 | 0.09 |
| South Georgia | 111 | 0.14 | 0.11 | 0.14 | 0.14 | 0.15 | 0.31 |

**Table 14.** Contribution (Q-values) of each population to assigned clusters (K = 3) using STRUCTURE for *Pareledone charcoti*

|  | Contribution to clusters | | | |
| --- | --- | --- | --- | --- |
| Cluster | n | 1 | 2 | 3 |
| Elephant Island | 350 | 0.34 | 0.34 | 0.32 |
| Peninsula | 11 | 0.35 | 0.29 | 0.36 |

**Table 15.** Contribution (Q-values) of each population to assigned clusters (K = 7) using STRUCTURE for *Pareledone turqueti*

|  |  | Contribution to clusters | | | | | |  |  |
| --- | --- | --- | --- | --- | --- | --- | --- | --- | --- |
| Cluster | n | 1 | 2 | 3 | 4 | 5 | 6 | 7 |  |
| Peninsula | 46 | 0.08 | 0.14 | 0.56 | 0.02 | 0.01 | 0.17 | 0.02 |  |
| Elephant Island | 93 | 0.24 | 0.49 | 0.89 | 0.02 | 0.02 | 0.12 | 0.02 |  |
| Signy Island | 9 | 0.03 | 0.18 | 0.29 | 0.05 | 0.06 | 0.35 | 0.03 |  |
| South Georgia | 182 | 0.01 | 0.04 | 0.04 | 0.37 | 0.06 | 0.10 | 0.38 |  |
| Shag Rocks | 125 | 0.01 | 0.03 | 0.03 | 0.04 | 0.76 | 0.09 | 0.05 |  |
| South Sandwich Islands | 1 | 0.01 | 0.08 | 0.44 | 0.11 | 0.04 | 0.30 | 0.02 |  |

**Table 16.** Directional migration estimates for *Pareledone turqueti* (null alleles adjusted)

|  |  | Source population | | | | | |
| --- | --- | --- | --- | --- | --- | --- | --- |
|  | Site | WAP | EAP | Elephant  Island | Signy  Island | South  Georgia | Shag  Rocks |
| Receiving population | WAP | - | 0.244 | 0.361 | 0.118 | 0.332 | 0.269 |
|  | EAP | 0.548 | - | 0.802 | 0.129 | 0.377 | 0.294 |
|  | Elephant Island | 0.454 | 0.497 | - | 0.174 | 0.464 | 0.410 |
|  | Signy Island | **0.423** | **0.334** | **0.746** | - | **0.735** | **0.448** |
|  | South Georgia | 0.269 | 0.203 | 0.604 | 0.128 | - | 1.000 |
|  | Shag Rocks | 0.286 | 0.178 | 0.483 | 0.179 | 0.715 | - |

Left column indicates where migrants travelled to; top row indicates where migrants originated from. Bold values are significant (based on 1000 bootstraps).

**Table 17.** Directional migration estimates for *Adelieledone polymorpha* (null alleles adjusted)

| Source population | | | | |
| --- | --- | --- | --- | --- |
|  | Site | Elephant  Island | Peninsula | South  Georgia |
| Receiving | Elephant Island | - | 1.00 | 0.405 |
|  | Peninsula | 0.686 | - | 0.336 |
|  | South Georgia | 0.265 | 0.184 | - |

Left column indicates where migrants travelled to; top row indicates where migrants originated from.

**Table 18.** Directional migration estimates for *Pareledone charcoti* (null alleles adjusted)

|  |  |  |  |  |  |  |  |
| --- | --- | --- | --- | --- | --- | --- | --- |
| \|  \|  \| Source population \|  \| \| --- \| --- \| --- \| --- \| \|  \| Site \| Elephant  Island \| Peninsula \| \|  \| Elephant Island \| - \| 0.570 \| \| Peninsula \| 1.00 \| - \|   Left column indicates where migrants travelled to; top row indicates where migrants originated from. |  |  |  |  |  |  |  |

**Table 19.** Contribution (Q-values) of each population of *P. turqueti* assigned clusters using STRUCTURE ordered by longitude

| Contribution to clusters | | | | | | | |
| --- | --- | --- | --- | --- | --- | --- | --- |
| Longitude | 1 | 2 | 3 | 4 | 5 | 6 | 7 |
| <55.3 | **0.33** | 0.24 | 0.21 | 0.03 | 0.03 | 0.15 | 0.02 |
| 55.3-58.75 | 0.16 | **0.60** | 0.05 | 0.02 | 0.02 | 0.13 | 0.02 |
| >58.75 | 0.06 | 0.11 | **0.60** | 0.03 | 0.01 | 0.18 | 0.02 |

**Table 20.** Contribution (Q-values) of each population of *P. turqueti* assigned clusters using STRUCTURE ordered by depth

| Contribution to clusters | | | | | | | |
| --- | --- | --- | --- | --- | --- | --- | --- |
| Depth (m) | 1 | 2 | 3 | 4 | 5 | 6 | 7 |
| <280 | 0.09 | **0.63** | 0.06 | 0.02 | 0.02 | 0.15 | 0.03 |
| 280-340 | **0.40** | 0.21 | 0.19 | 0.03 | 0.02 | 0.13 | 0.01 |
| >352 | 0.11 | 0.04 | **0.63** | 0.02 | 0.01 | 0.17 | 0.01 |

**Table 21.** Statistical power for detecting levels of population differentiation (F_ST_) by means of Chi^2^ and Fisher’s exact tests calculated using POWSIM. Power is expressed as the proportion of simulations providing statistical significance at the 0.05 level.

| True F_ST_ | *P. turqueti*  (10 loci, 6 populations) | | 1. *polymorpha*   (9 loci, 3 populations) | | *P. charcoti*  (10 loci, 2 populations) | |
| --- | --- | --- | --- | --- | --- | --- |
|  | Chi^2^ | Fisher | Chi^2^ | Fisher | Chi^2^ | Fisher |
| 0.0010 | 0.9660 | 0.9420 | 0.7190 | 0.7500 | 0.4850 | 0.1130 |
| 0.0025 | 1.0000 | 1.0000 | 1.0000 | 1.0000 | 0.8280 | 0.3490 |
| 0.0050 | 1.0000 | 1.0000 | 1.0000 | 1.0000 | 0.9800 | 0.7270 |
| 0.0100 | 1.0000 | 1.0000 | 1.0000 | 1.0000 | 1.0000 | 0.9820 |
| 0.0200 | 1.0000 | 1.0000 | 1.0000 | 1.0000 | 1.0000 | 1.0000 |
| 0.0250 | 1.0000 | 1.0000 | 1.0000 | 1.0000 | 1.0000 | 1.0000 |
| 0.0500 | 1.0000 | 1.0000 | 1.0000 | 1.0000 | 1.0000 | 1.0000 |

**Fig. 1.** Evaluation of the locations of five potential barriers to gene flow among samples of the octopus *P. turqueti* inhabiting the Southern Ocean, as identified using BARRIER v. 2.2 (Manni et al. 2004). Principal barriers (red lines) among samples (black dots) are identified according to the level of genetic differentiation (F_ST_) among pairs of samples (with barrier 1 representing the location of the strongest limit to gene flow, followed by barrier 2, then barrier 3, *etc*.). Numbers and width of barrier indicate bootstrap support (most prominent barrier highlighted bold) for the location of each potential barriers location. PEN, Western and Eastern Antarctic Peninsula; ELE, Elephant Island; SIG, Signy Island; SOG, South Georgia; SHR, Shag Rocks (see Figure 1 for sample locations and Table 3 for a matrix of estimates of pairwise F_ST_ among samples).


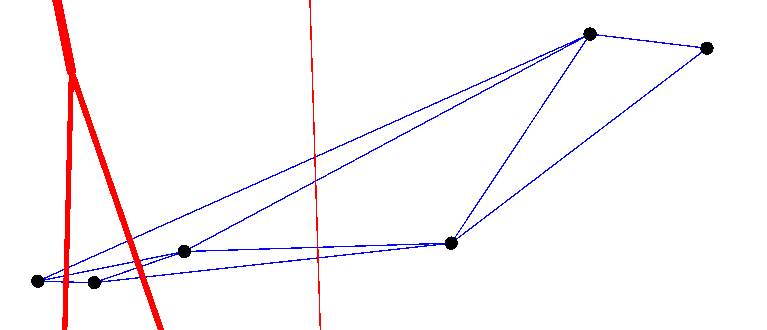

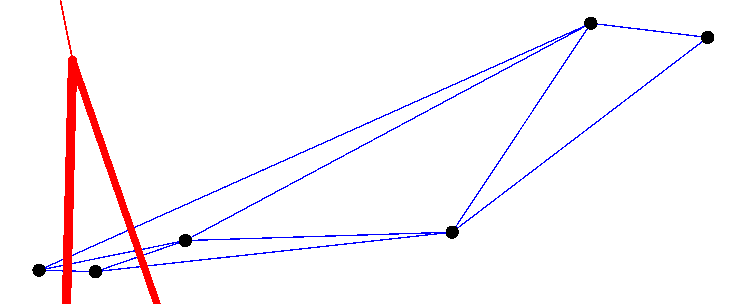

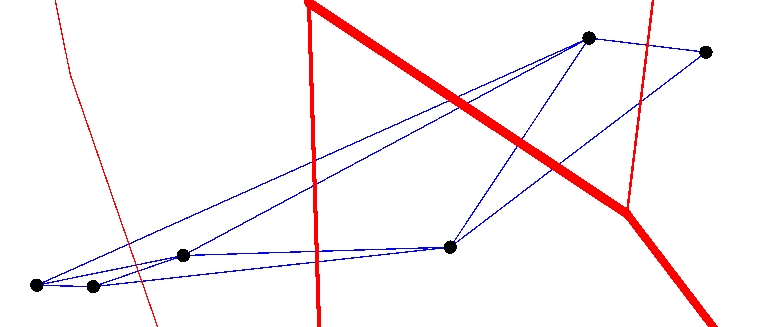

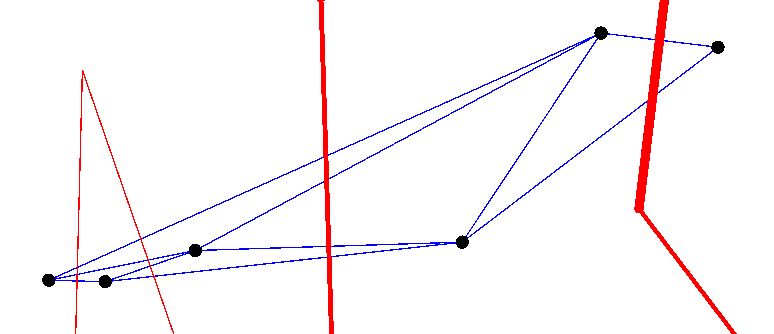

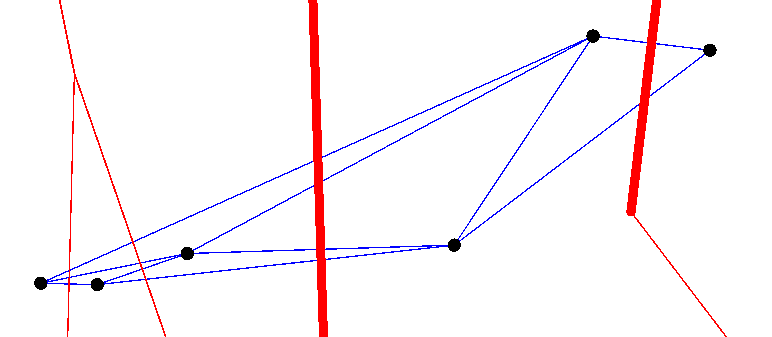


Barrier number

5 4 3 2 1

SHR

SOG

1

16

**100**

35

PEN

84

ELE

SIG

34

**35**

1

1

16

14

**49**

1

3

47

2

55

45

**91**

2

4

**53**

40
